# Dissemination of Evidence by Cochrane Public Health Europe in German-Speaking Countries: An Online Stakeholder Survey

**DOI:** 10.3389/ijph.2022.1605265

**Published:** 2022-12-16

**Authors:** Ursula Griebler, Christina Kien, Karina K. De Santis, Jan Stratil, Annegret Borchard, Thomas L. Heise

**Affiliations:** ^1^ Department of Evidence-Based Medicine and Evaluation, Danube University Krems, Krems an der Donau, Austria; ^2^ Cochrane Austria, Krems an der Donau, Austria; ^3^ Department of Prevention and Evaluation, Leibniz Institute for Prevention Research and Epidemiology-BIPS, Bremen, Germany; ^4^ Institute for Medical Information Processing, Biometry and Epidemiology (IBE), Chair of Public Health and Health Services Research, Ludwig Maximilian University of Munich, Munich, Germany; ^5^ Pettenkofer School of Public Health, Munich, Germany; ^6^ Cochrane Switzerland, Center for Primary Care and Public Health (Unisanté), University of Lausanne (UNIL), Lausanne, Switzerland; ^7^ Institute for Public Health and Nursing Research, Health Sciences Bremen, University of Bremen, Bremen, Germany

**Keywords:** public health, knowledge translation, Cochrane reviews, dissemination, evidence use

## Abstract

**Objectives:** To investigate the reach and impact of “Infomails”, email summaries of Cochrane reviews in German, regularly disseminated by Cochrane Public Health Europe (CPHE) to stakeholders in Austria, Germany and Switzerland.

**Methods:** We analysed email campaign reports from 15 Infomails delivered until November 2020. Furthermore, we invited all previous Infomail recipients to participate in an online survey on the impact and perceptions regarding our Infomails in November 2020. We analysed the results using descriptive statistics.

**Results:** The Infomails’ open rate ranged from 10.9% to 39.3% (median 26.0%), and the median click rate on the embedded links was 28.0% (range 8.6–53.8%), highest for nutrition and prevention topics. Out of 1259 recipients, 267 (21.2%) completed our survey. Infomails were most used in discussions, writing reports or statements, for policy or strategy development or programme or guideline development. Persons who remembered receiving Infomails rated them as useful, comprehensible or informative.

**Conclusion:** Infomails summarising recent Cochrane reviews were considered useful for the daily work of public health stakeholders in German-speaking countries. Regular targeted messaging may increase the perceived usefulness.

## Introduction

*Knowledge translation* is a term commonly used to address the knowledge-to-action gap between existing research knowledge and its application in public health practice and policy settings [1]. Generally, knowledge translation refers to the effective use of knowledge to benefit all aspects of health [2]. The World Health Organisation (WHO) defines knowledge translation as “the synthesis, exchange, and application of knowledge by relevant stakeholders to accelerate the benefits of global and local innovation in strengthening health systems and improving people’s health” [3]. The use of evidence to inform public health policy and practice decisions has been increasingly viewed as important since the term *evidence-based public health* was coined over 20 years ago [4, 5]. Barriers to using research evidence in health decision-making include poor access to good-quality, relevant research, lack of timeliness, and a lack of considering the needs of practitioners and policymakers regarding the presentation format of research findings [6, 7]. Facilitators for policymakers to use evidence can be personal contact between researchers and policymakers, the provision of summaries and clearly highlighting key messages [6–8]. Translating such knowledge can be facilitated when the accessible evidence is high quality, understandable and appropriately disseminated to relevant stakeholder groups [9, 10]. Ideally, end-users’ evidence needs are already known before any knowledge translation activity begins: a recent survey of social security and insurance medicine professionals in the European Union showed that synthesised evidence based on primary research, as reported in guidelines and systematic reviews, is more often needed for their work than primary study evidence alone [11].

Dissemination methods include “push” activities undertaken by research organisations to actively disseminate research evidence (e.g., *via* email or social media), “pull” activities undertaken by decision-makers to access and apply research evidence, and “exchange” activities to build and maintain relationships between researchers and decision-makers [12].

Cochrane is an international, not-for-profit network of health experts and users dedicated to improving health outcomes worldwide [13]. Cochrane supports knowledge translation by 1) contributing high-quality systematic reviews on healthcare interventions to inform health decision-making and 2) disseminating the evidence from such reviews to relevant stakeholders in policy settings [14]. Cochrane Public Health (CPH) is one of over 50 review groups within Cochrane [15] and was established to specifically address public health interventions. Cochrane Public Health Europe (CPHE) is a regional, German language–based CPH satellite that regularly performs dissemination activities in line with the Cochrane Knowledge Translation Framework Theme Two: “Packaging, push and support to implementation: Ensuring our users receive and can act on our reviews and products” [16]. As part of the CPHE dissemination activities, a subgroup of CPHE members formed the CPHE dissemination team that regularly sends out German-language email summaries of CPH reviews called “Infomails” to handpicked, review-specific public health stakeholders in Austria, Germany and Switzerland. The Infomails’ overarching aim is to inform potentially interested stakeholders of newly available CPH reviews and facilitate their translation into decision- and policy-making processes. We have developed a standardised procedure for producing and disseminating the Infomails and started sending out CPHE Infomails in autumn 2017. When a new CPH review is about to be published, the CPHE dissemination team consisting of representatives of the participating institutions, i.e., approximately five CPHE researchers first discuss whether the results will be of interest to stakeholder groups in Austria, Germany and Switzerland. In our decision we balance the effort of producing the Infomails and stakeholder lists and the anticipated level of interest of the recipients. Reviews with e.g., very low strength of evidence or “empty” reviews and little anticipated practical information for policymakers or practitioners are not disseminated. Once the decision to send an Infomail on the respective CPH review is made, the plain language summary is translated into German using a standardized procedure including checks by Cochrane Germany who coordinate the German translation work and published on the “Cochrane Kompakt” website [17]. Based on an Infomail template that we developed collaboratively, one person from the CPHE dissemination team is drafting the Infomail text first and receives feedback from the other team members who also take care that the text is prepared according to the Cochrane dissemination checklist [18]. We further developed separate review-specific stakeholder lists by searching the internet for organisations and institutions related to the review topic and named persons from our own professional networks for Austria, Germany and Switzerland, thus ensuring only potentially interested persons receive our Infomail. We specifically focused on political decision-makers, public health authorities, supporting bodies for political decision-makers and practitioners. Infomails are sent in irregular intervals, depending on the timing of the publication of a CPH review. During the first 3 years of sending Infomails to stakeholders, we rarely received direct feedback, and our approach was very resource-intensive. Therefore, we reached out to our stakeholders to investigate the following aims:

1) To analyse the Infomails’ reach and impact
2) To get direct feedback on the Infomail format to tailor our dissemination activities to our recipients’ needs
3) To investigate the stakeholders’ preferred methods of informing themselves about research evidence
4) To describe in general how the recipients of our dissemination activities are using evidence in public health decision-making

## Methods

### Study Design

This multi-method study consisted of the analysis of the Infomail email campaign reports and a cross-sectional online survey among all previous Infomail recipients (i.e., intended full survey) and took place in Austria, Germany and Switzerland in November 2020. The study reporting is in accordance with the STrengthening the Reporting of OBservational studies in Epidemiology (STROBE) statement for cross-sectional studies [19] and the Checklist for Reporting Results of Internet E-Surveys (CHERRIES) [20].

We developed a study protocol (available on request) where we defined the aims and research questions, outcome measures and planned analyses before the study conduct.

### Email Campaign Reports

We used Mailchimp^®^ to send all Infomails to separate audiences in Austria, Germany and Switzerland. The Infomails’ reach was measured using the email campaign reports from all Infomails delivered up to November 2020. In each Infomail we provided links to both the German plain language summary on the “Cochrane Kompakt” platform (https://www.cochrane.org/de/evidence) and the original full-text review in English in the Cochrane library (https://www.cochranelibrary.com/). We used the percentage of successful deliveries (i.e., the proportion of successfully delivered emails in relation to sent emails), the open rate (i.e., the proportion of opened emails in relation to all successfully delivered emails) and the click rates per unique opening and successful delivery (i.e., the proportion of clicks on embedded links in relation to opened or successfully delivered emails, respectively) as outcome measures.

### Online Survey Among Previous Infomail Recipients

#### Survey Development

An online survey was developed to measure the use of evidence in general, the Infomails’ impact and further suggestions for their improvement. The survey comprised three parts: Part 1 measured types of information use and the frequency and purpose of information used by stakeholders as well as preferred ways to inform themselves about research results in the context of their work. Part 2 assessed their perceptions regarding the Infomails’ format and impact on their work, and asked for feedback for improvement. Part 3 covered demographic questions about the respondents’ characteristics and their area of work and expertise.

Part 1 of the survey was developed based on the previous literature on evidence use [21–23]; parts 2 and 3 were developed based on our own expertise among the research team.

The questionnaire was developed in German. The survey’s face validity was confirmed among the research team. We distributed the online survey using the REDCap 10.6.26 platform and pilot-tested it with five research colleagues uninvolved in the study development, who also confirmed the survey’s face validity and easiness to handle the online questionnaire. A detailed description on the survey development methods is included in the Supplementary Material. The survey questions are available from the corresponding author on request.

#### Survey Participants

The online survey’s target population included all previous recipients of at least one Infomail since their inception in November 2017 up to November 2020 (i.e., full survey). Recipients were stakeholders from Austria, Germany or Switzerland who we searched for (e.g., through organisational websites, personal contacts) and hand-picked specifically for each review topic. We considered stakeholders from the following stakeholder groups:

• Political decision-making body: planning and development of strategies, legislation
• Administration, authority: planning and development of strategies or their implementation
• Supporting body for political decision-makers or policy advice
• Practice: planning and operational implementation of programmes and projects
• Associations and interest groups
• Researchers
• Industry
• Others (e.g., journalists)

#### Survey Procedure

We sent an online survey invitation per email to 1380 individuals in total: 451 from Germany, 466 from Austria and 463 from Switzerland. The first invitation to the voluntary survey was sent on 11 November 2020 and a reminder on 23 November 2020. No incentives for participating in the survey were offered. The items were not randomized for the participants as the survey followed a logical order.

#### Statistical Analysis

Based on the final survey data downloaded from REDCap, we performed a plausibility check and recoded several items that were reverse coded for data analysis. Furthermore, one researcher read through all qualitative answers to the open-ended questions and developed further answer categories inductively. A second researcher checked these new categories for correctness. We used descriptive statistics and performed all analyses with the statistical software package IBM SPSS Statistics for Windows Version 27.0. No weighting of items or propensity scores have been used to adjust for possible non-representativeness of the sample.

#### Ethical Issues

The Ethics Committee of the Danube University Krems, Austria declared non-competence for this cross-sectional online survey, because surveys involving adults not belonging to a vulnerable group need no ethics approval. All participants gave their consent voluntarily, having been informed of the purpose and the length of the study, ensuring at all times the confidentiality of the data and the anonymity of the participants. The personal email address used for sending out the link to the survey was deleted for the analysis process. This study was performed in accordance with the 1964 Helsinki declaration and its later amendments [24].

## Results

### Email Campaign Reports

In total, 15 Infomails disseminated between November 2017 and December 2020 were included in this analysis. These covered a diverse range of topics: prevention (*n* = 1), nutrition (*n* = 8), physical activity (*n* = 1), social support and social networks (*n* = 1), natural and physical environment (*n* = 1), economic interventions (*n* = 2) and COVID-19 (*n* = 1).

In total, 3343 emails were sent to 1380 recipients, with some receiving more than one Infomail. Of all sent emails, 96.1% (*n* = 3207) were successful deliveries.

#### CPHE Infomails’ Reach and Impact

Across the deliveries of the 15 Infomails, the number of recipients in all three countries ranged from 86 to 429, with a median of 177 recipients. The Infomails’ open rate ranged from 10.9% to 39.3%, with a median of 26.0% (see Table 1). Of the five with the highest open rates, three were on nutrition-related topics: nutritional labelling (Infomail No. 3) with a 39.3% open rate, environmental interventions to reduce sugar-sweetened beverage consumption (Infomail No. 6) with a 32.7% and sugar taxation (Infomail No. 10) with a 30.3% open rate.

**TABLE 1:** Results of the Infomail campaign reports for all three countries, per Infomail (Evaluation of Cochrane Public Health Europe Infomails, Austria/Germany/Switzerland, 2020).

Infomail number	Short title of cochrane review	Topic	Cochrane review identification number	Date the Infomail was sent	Number of Infomail recipients	Successful deliveries (*n*)/%		Opens (n)/open rate per successful delivery (%)		Clicks (n)/click rate per unique opening (%)/click rate per successful delivery (%)		
1	Welfare-to-work interventions	Economic interventions	CD009820	November 2017	86	75	87.2%	20	26.7%	2	10.0%	3.6%
2	Unconditional cash transfers	Economic intervention	CD011135	December 2017	90	87	96.7%	24	27.6%	2	8.3%	3.2%
3	Nutritional labelling	Nutrition	CD009315	March 2018	149	145	97.3%	57	39.3%	23	40.4%	26.1%
4	Multiple risk behaviours	Prevention	CD009927	October 2018	177	172	97.2%	61	35.5%	26	42.6%	23.4%
5	Ambient air pollution	Natural and physical environment	CD010919	May 2019	96	96	100%	19	19.8%	2	10.5%	2.6%
6	Environmental interventions to reduce SSB consumption	Nutrition	CD012292	June 2019	345	333	96.5%	109	32.7%	47	43.1%	21.0%
7	Iodine fortification	Nutrition	CD010734	July 2019	101	100	99.0%	26	26.0%	14	53.8%	18.9%
8	Altering availability of food products	Nutrition	CD012573	September 2019	429	419	97.7%	93	22.2%	34	36.6%	10.4%
9	Fortification (5 reviews)	Nutrition	CD010697	November 2019	132	129	97.7%	19	14.7%	2	10.5%	1.8%
			CD010187									
			CD010068									
			CD012150									
			CD009902									
10	Taxation of sugar	Nutrition	CD012333	April 2020	311	294	94.5%	89	30.3%	36	40.4%	17.6%
11	Video calls to reduce social isolation	Social support and social networks	CD013632	June 2020	356	341	95.8%	98	28.7%	37	37.8%	15.2%
12	Travel‐related control measures	COVID-19	CD013717	September 2020	203	195	96.1%	50	25.6%	14	28.0%	9.7%
13	Interventions to reduce sedentary behaviour	Physical activity	CD012554	October 2020	361	344	95.3%	60	17.4%	14	23.3%	4.9%
14	Taxation of the fat content of foods	Nutrition	CD012415	October 2020	354	339	95.8%	68	20.1%	11	16.2%	4.1%
15	Wheat flour fortification with iron	Nutrition	CD011302	December 2020	153	138	90.2%	15	10.9%	2	13.3%	1.6%
	**Median**				**177**	**172**	**96.5%**	**57**	**26.0%**	**14**	**28.0%**	**9.7%**
	**Sum**				**3343**	**3207**		**808**		**266**		

Abbreviations: SSB, sugar-sweetened beverages.

The bold values are the median and the sum.

In general, the median click rate per successful delivery was 9.7% (range 1.6%–26.1%), meaning that on average 9.7% of successful recipients clicked on at least one link within the Infomail. The median click rate per unique opening was 28.0%, where recipients clicked on at least one embedded link in the Infomail (range 8.3%–53.8%; median clicks 14, range 2–47), most often on the link to the plain language summary in German (median click rate 21.4%) or to the full text in English (median click rate 5.9%) (see Table 1 and Supplementary Table S1).

**FIGURE 1:**
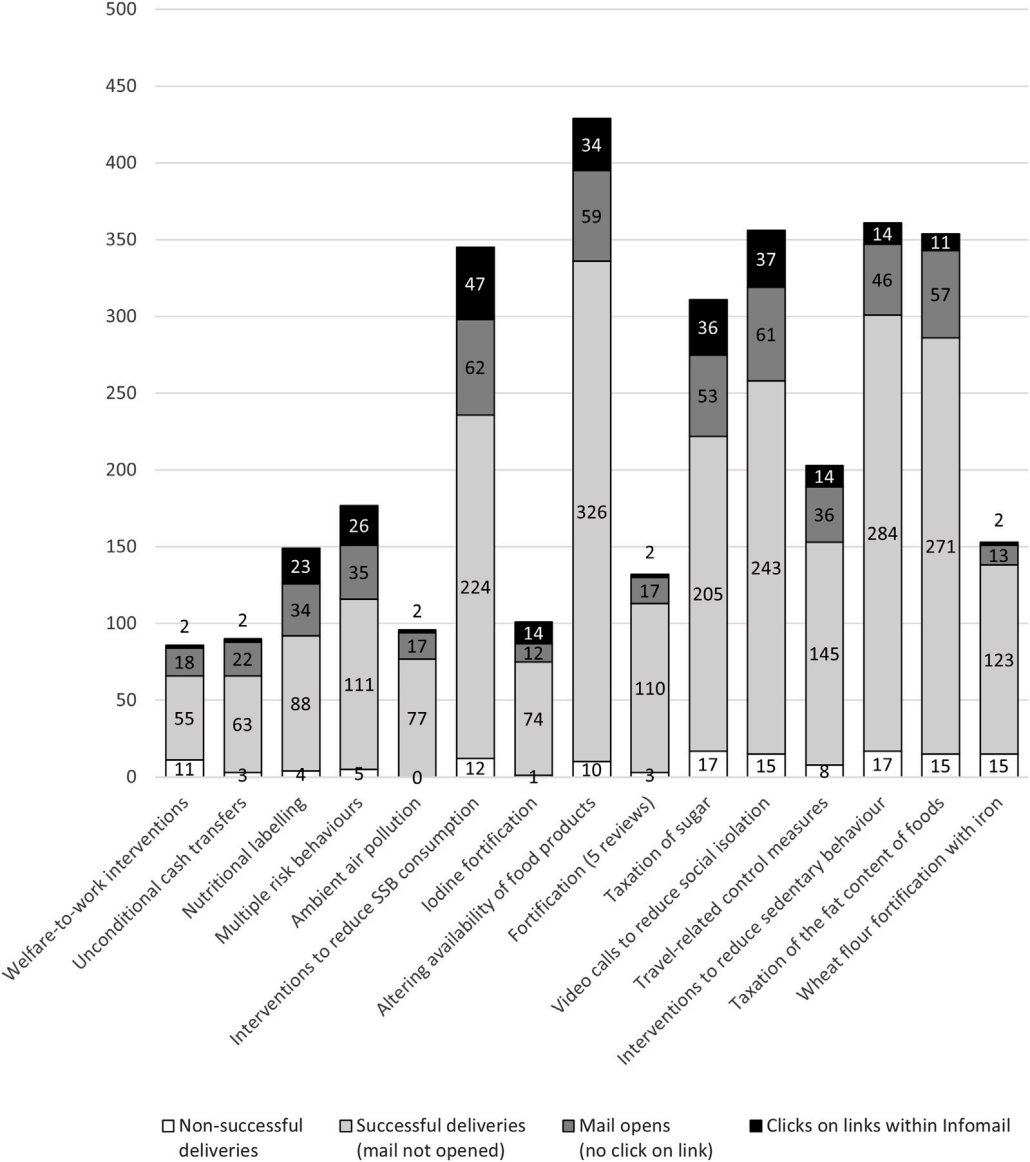
Results on the delivery, opens and clicks of the 15 Infomails campaigns, absolute numbers (Evaluation of Cochrane Public Health Europe Infomails, Austria/Germany/Switzerland, 2020).

Figure 1 shows the absolute numbers of deliveries, email openings and clicks on links for all 15 Infomails. The Infomails showed heterogeneous results across the three German-speaking countries (see Supplementary Table S2): the median number of recipients per Infomail was higher in Switzerland (*n* = 74) than in Germany or Austria (*n* = 47 and 51, respectively). In Switzerland, around one-third of recipients opened the Infomail (median open rate 30.4%), while in Austria and Germany the median open rate was 22.9% and 24.1%, respectively. However, the median click rate was highest in Austria (35.0% per unique opening and 9.2% per successful delivery), followed by Germany (30.8% and 7.1%) and Switzerland (18.2% and 4.3%).

### Online Survey Among Previous Infomail Recipients

#### Participation Rate and Description of Participants

For 6.6% (91/1380), the email message was undeliverable. Furthermore, 30 recipients indicated non-participation in the survey, as their respective job had changed within or across organisations. Therefore, out of 1259 Infomail recipients, 267 (21.2%) participated in our survey and 226 (84.6%) completed the survey in full.

Approximately half of the respondents (53.7%) were female, 58.6% were at least 50 years old, and 67.0% held senior managerial positions with staff responsibility (see Supplementary Table S3). Over half of the participants had 10+ years of work experience in their respective field. The participants comprised stakeholders from research institutions (26.0%), organisations involved with planning and operational implementation of programmes and projects (22.5%), associations and interest groups (19.8%), and administrative institutions (19.4%). 45.3% resided in Austria, 33.0% in Switzerland and 21.7% in Germany, resulting in an overrepresentation of Austrian participants and an underrepresentation of German participants in comparison to the Infomail recipient population (approximately one-third per country). About three-quarters of the participants (72.6%) were familiar with Cochrane’s work. Stakeholders from associations and interest groups (62.2%) as well as those from administrative institutions and authorities (61.4%) were less familiar with Cochrane.

#### Usage of the Infomails

In general, the respondents used the Infomails’ content in contributing to discussions in their work context (39.3%, 96/244), writing reports or statements (25.4%, 62/244) and as information bases for policy or strategy development (22.5%, 55/244) or for program (20.5%, 50/244) or guideline development (13.1%, 32/244) (Supplementary Table S4). One-third (35.7%, 87/244) could not remember having received any Infomail, and 4.5% (11/244) mentioned that Infomails had no relevance to their daily work. Those having received only one Infomail reported more often (43.0%, 61/142) that they could not remember having received an Infomail than those having received Infomails twice (30.3%, 10/33) or more than twice (17.8%, 16/90). 66.3% (102/154) of respondents that remembered having received Infomails indicated that they considered the Infomails rather or very helpful for their daily work. Of those who reported using the Infomail’s content (61.3%, 149/243), 80.5% (120/149) indicated that they used it for more than one purpose.

#### Feedback Regarding the Infomails

**FIGURE 2:**
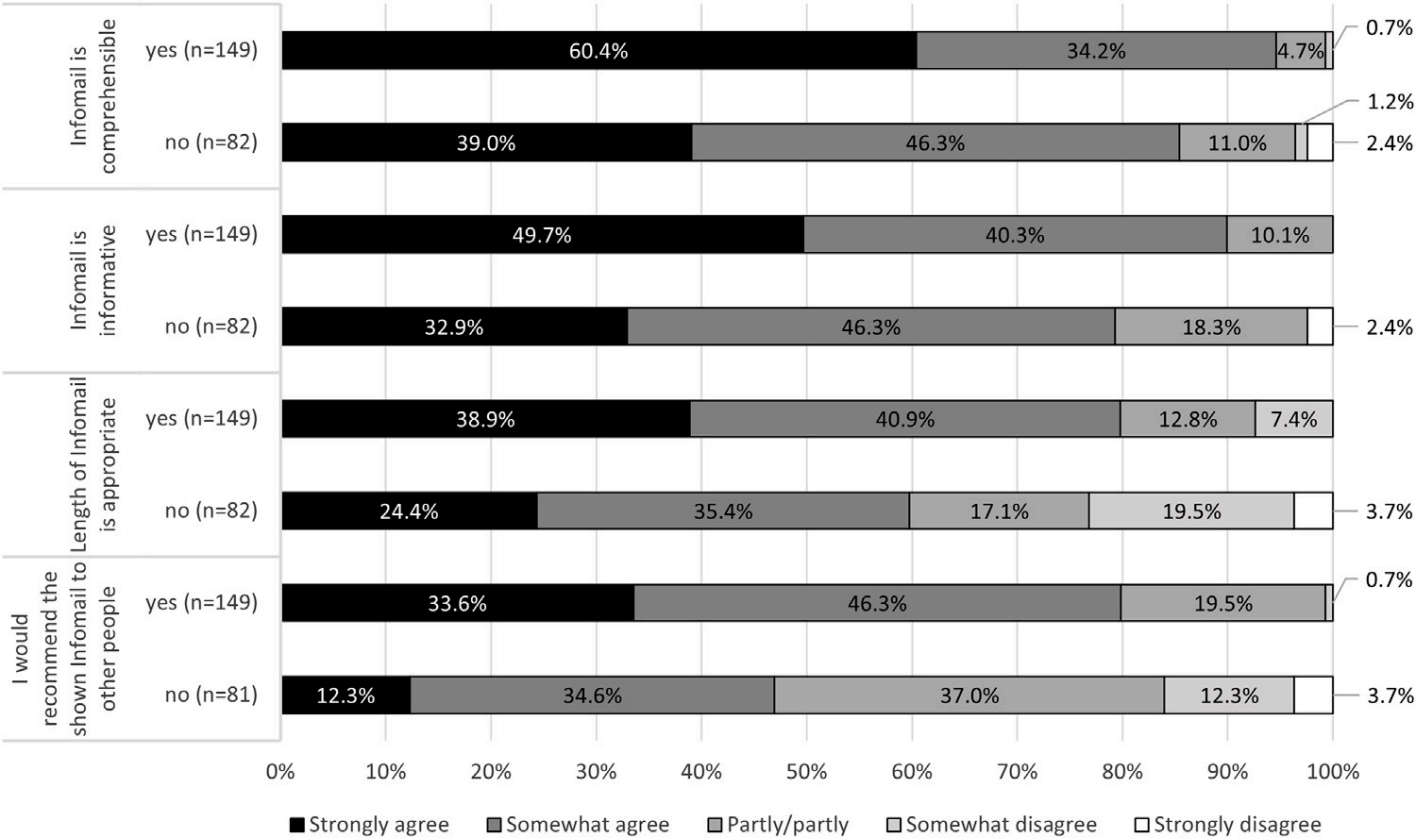
Feedback regarding the Infomail, separate for persons remembering ever having received an Infomail (yes) vs. not remembering (no) (Evaluation of Cochrane Public Health Europe Infomails, Austria/Germany/Switzerland, 2020).

An example Infomail was shown within the survey, and participants could provide feedback on its layout and content. Generally, those who remembered receiving an Infomail rated it more as informative and comprehensible as well as appropriate in length and agreed more with the statement that they would recommend it to other people compared to those not remembering having received an Infomail in the past (Figure 2). Respondents who disagreed (partly, somewhat or strongly) that the Infomail’s length was appropriate mentioned in general (98.4%, 60/61) that it was much or rather too long. The word count of the main text of the example Infomail was 297 words. The average word count of all 15 Infomails was slightly higher with 283 words.

#### Feedback Regarding Improvement of the Infomail Design

Feedback regarding improvement of the Infomail design was provided by 48 out of 267 respondents (18.0%). 39.6% suggested adding subheadings to improve clarity or visual structure and proposed shortening the Infomails. Approximately one-fifth recommended highlighting the most important information *via* infoboxes, figures or tables (22.9%) or bold or coloured text (22.9%). Furthermore, 18.8% suggested adding a teaser or main message at the beginning; 14.6% stressed that the validity or practical relevance of the context should be checked and reported.

#### Preferred Form of Disseminating Cochrane Public Health Review Results

When asked about the preferred form of information dissemination, almost all respondents (95.9%, 209/218) stated their interest in a topic-specific newsletter for the latest CPH reviews. 21.6% (47/218) indicated their interest in dissemination *via* Facebook, and 17.9% (39/218) *via* Twitter. The only remarkable age difference was observed regarding dissemination *via* Facebook: while 33.7% (29/86) of respondents aged 50 or younger would prefer disseminating the results *via* Facebook, only 13.4% (17/127) aged 51 or over would prefer this dissemination strategy. The most requested topics were prevention (78.9%, 183/232), social support and social networks (49.6%), physical activity (48.3%, 115/232) and nutrition (46.6%, 108/232).

#### Preferred Methods to Inform Themselves About Research Evidence

**FIGURE 3:**
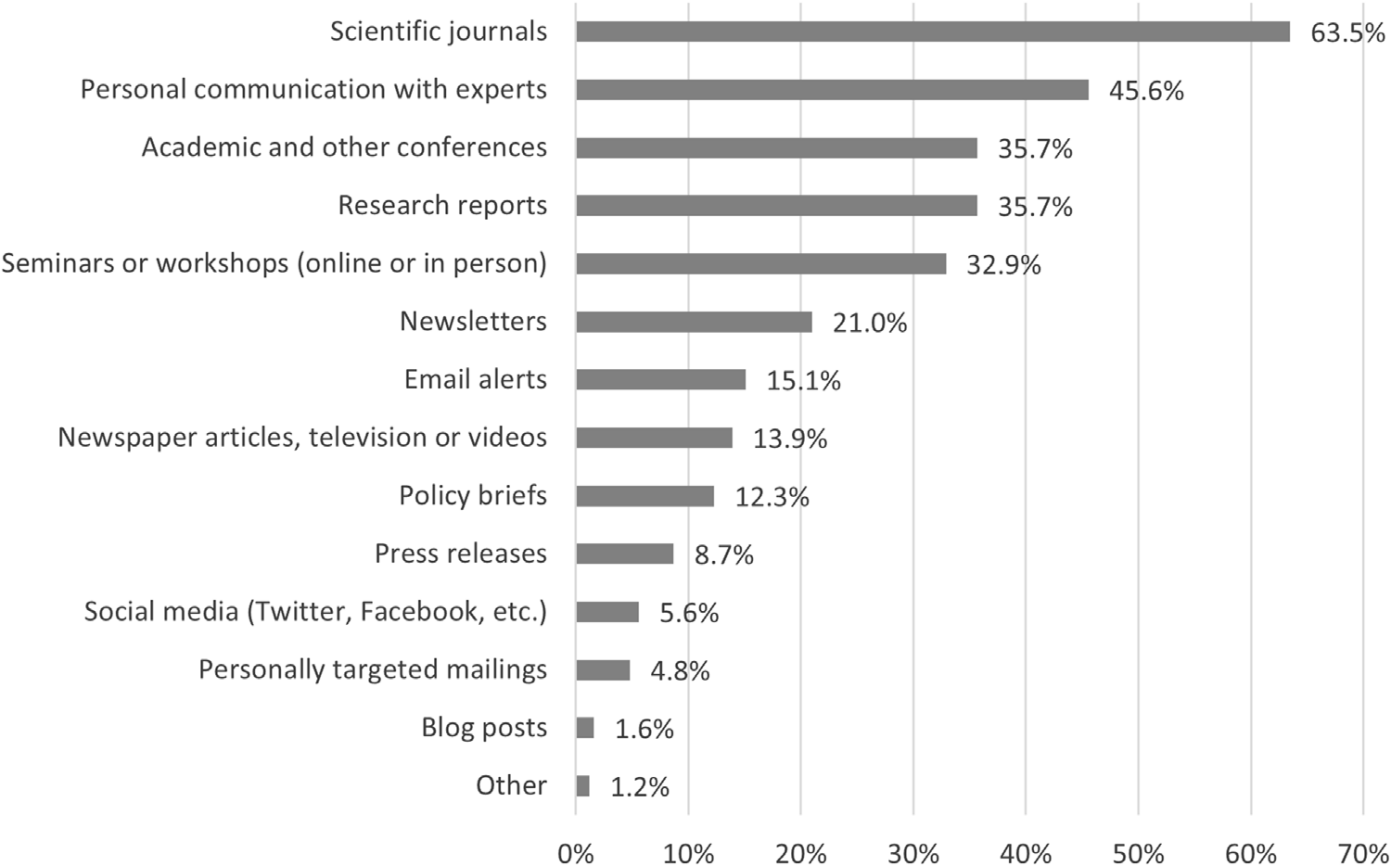
Preferred way to get informed about research results for work purposes (*n* = 252) (Evaluation of Cochrane Public Health Europe Infomails, Austria/Germany/Switzerland, 2020).

When asked about their preferred way to get informed about research results in the course of their work, 63.5% (160/252) of respondents indicated using scientific journals (see Figure 3), the preferred method among the subgroup of stakeholders working in the research area (96.6%, 57/59). Personal communication with experts was the second most popular way to receive information (45.6%, 115/252) and the preferred strategy of stakeholders from practice (56.9%, 29/51), administrative institutions (68.2%, 30/44) or political bodies (62.5%, 5/8). Further ways to get informed were research reports (35.7%, 90/252) and research presented at academic and other conferences (35.7%, 90/252) and seminars or workshops (32.9%, 83/252). Newsletters and personally targeted mailings were mentioned less often (21.0%, 53/252 and 4.8%, 12/252).

#### Use of Evidence in Daily Work and Decision-Making

Overall, 71.5% of respondents (191/267) indicated that they would use five, six or seven different information types in their work. Almost all (99.3%) indicated that they would use data, statistics and reports in their work, more than half (56.5%) on a weekly or daily basis (Table 2). The vast majority (94.8%) reported using academic research evidence, almost six out of ten (59.2%) on a weekly or daily basis. 87.6% indicated that they were seeking expertise and advice, 45.1% on a weekly or daily basis. Approximately one-third of the respondents (34.1%) indicated the usage of other information types, such as traditional and social media (9.7%), discussions with colleagues or stakeholders (6.4%) or grey literature (5.2%). Most information types were rated as very or rather useful by most respondents, except for traditional and social media where almost 40% were unsure about this information type’s usefulness for their work (Table 2).

**TABLE 2:** Work-related usage, frequency of usage and usefulness of different information types (Evaluation of Cochrane Public Health Europe Infomails, Austria/Germany/Switzerland, 2020).

	Usage in general		Frequency of usage, in %						Usefulness of the used information types, in %					
	n	%	Daily	Weekly	Monthly	Quarterly	Less than quarterly	n	Very useful	Rather useful	Partly, partly	Rather unuseful	Not at all useful	n
Data, statistics and reports	265	99.3	15.3	41.2	27.1	12.9	3.5	255	51.6	29.8	17.7	0.8		248
Academic research evidence	253	94.8	25.5	33.7	24.7	10.7	5.3	243	46.6	35.7	16.4	1.3		238
Expertise and advice	234	87.6	8.9	36.2	27.2	20.5	7.1	224	49.1	35.9	13.6	0.5	0.9	220
Practice guidelines	231	86.5	5.4	25.1	39.5	19.7	10.3	223	39.8	37.5	21.8	0.5	0.5	216
Policies/legislation and legal information	222	83.1	14.9	28.8	25.6	16.7	14.0	215	33.5	34.9	26.3	5.3		209
*Other information sources*	91	34.1	37.8	24.4	22.2	8.9	6.7	90	43.8	34.8	19.1	1.1	1.1	89
Traditional and social media	26	9.7	69.2	15.4	3.8	7.7	3.8	26	23.1	38.5	38.5			26
Discussions with colleagues or stakeholders	17	6.4	11.8	23.5	35.3	23.5	5.9	17	68.8	12.5	18.8			16
Grey literature	14	5.2	7.7	38.5	46.2		7.7	13	53.8	38.5	7.7			13
Specialist literature	9	3.4		44.4	55.6			9	11.1	77.8	11.1			9
Own research data, surveys	5	1.9	20.0		20.0	20.0	40.0	5	80.0	20.0				5
Online scientific databases	2	0.7	50.0	50.0				2	100.0					2

Abbreviations: n = sample size, % = percentage.

## Discussion

In this study, we evaluated the reach and impact of targeted email messages (called “Infomails”) comprising German-language summaries of recent CPH review results on stakeholders in Austria, Germany and Switzerland.

Overall, the Infomails’ median open rate was 26.0% (range 10.9%–39.9%), comparable to the average open rates of 27.4% reported by Constant Contact (an email marketing platform similar to Mailchimp, as used in our study) for the health and wellness business type [25]. Few comparable studies exist in the literature: Woodruff et al. [26] sent biweekly emails containing links to a website summarising evidence on abortion facility regulation and reported an overall rate of 36% (95% CI 29–42%) for opening at least one out of five emails.

The median click rate on links provided within our Infomails was 28.0% (range 8.6–53.8%) and differed depending on the Infomail’s content and topic. The three most successful Infomails with open and click rates over 30% and 40%, respectively, were two on nutrition-related topics and one on prevention.

Targeted and tailored messaging is a commonly used knowledge translation strategy to promote evidence-informed decision-making. It counts among the “push” activities undertaken by research organisations to disseminate research evidence. Further knowledge translation strategies can be categorised into “pull” activities undertaken by decision-makers to access and use research evidence and “exchange” activities to build and maintain relationships between researchers and decision-makers [27–29]. Dobbins et al. [30] showed in a randomised controlled trial comparing three knowledge translation and exchange strategies that tailored, targeted messages plus website information materials can be an effective strategy for facilitating evidence-informed decision-making [30]. In a systematic review, LaRocca et al. [31] concluded that passive knowledge translation strategies (i.e., access to registries of pre-processed research evidence or print materials) were less effective in promoting evidence-informed decision-making among public health decision-makers.

Evidence summaries like those used in our Infomails are likely easier to understand than complete systematic reviews. However, their ability to increase the use of systematic review evidence in policymaking is unclear [32].

In our cross-sectional survey among previous Infomail recipients, we asked about the information types used in their daily work. Almost all respondents indicated using data, statistics and reports as well as academic research evidence in their work, more than half on a weekly or daily basis. Seeking expertise and advice and using practice guidelines or policies/legislation and legal information was also indicated by over 80%, but the frequency of use was lower. Other information types such as traditional and social media were used less often (9.7%), but among those who did, most frequently with almost 70% of daily users. Our results do not correspond with those of previous studies, such as by Zardo et al. [22], who showed research evidence was the information type used less commonly and least frequently, and internal data and reports was the information type used most commonly and frequently among two government public health agencies to inform their day-to-day decision-making. However, our survey respondents’ work fields differed substantially, as almost half work in either research or practice. In a survey by Oliver and de Vocht [23] among public health policymakers and evidence producers working in a large UK city, local data was the most frequently used type of data (95%), and between 40% and 60% indicated using research-derived evidence. The most frequently mentioned sources were government websites (84%), followed by National Institute for Health and Care (NICE) guidelines (70%); “experts” and “other people” were both chosen by over 70% of respondents [23]. Conversely, in our study around two-thirds of respondents indicated scientific journals as the preferred way of getting informed about research evidence. Similar to Jacob et al.’s findings [21], the preferences for how to receive research evidence varied with work field in our study, with almost all researchers indicating a preference for scientific journals, whereas personal communication with experts was the top choice for administrative and political bodies as well as practice. Furthermore, most commonly, researchers disseminate their research evidence through academic journals, which results in a mismatch between practitioners’ and policymakers’ preferences for receiving research evidence on one hand and the dissemination strategies used by researchers on the other [12].

The assessment of the Infomails differed depending on the respondent’s remembrance of ever having received one. Those who remembered having received an Infomail rated the example Infomail as comprehensive and informative and would recommend it to other people. Also, Woodruff et al. [26] concluded that repeated mailings may increase the effectiveness.

Other non-randomised studies using digital technology in knowledge translation activities were done on clinical topics and have mainly investigated familiarity with research interventions [33], uptake of evidence into practice [34] and intention to use evidence in patient care [35].

### Study Limitations

German stakeholders’ participation rate was lower than that of respondents from Austria, possibly because the survey invitation was sent from an Austrian researcher. Thus, the generalisability of the results may be limited. The number of Infomail recipients differed according to the review topic and from country to country, depending on the time resources and number of personal contacts. One further reason for the higher click and open rates of nutrition-related topics could be that two persons who were responsible for preparing the stakeholder lists for the Infomails are nutritional scientists and have more personal contacts with stakeholders in this field. Persons may be more likely to open emails from people they know.

The extent to which our sample was representative of the target population (i.e., all previous receipients) is difficult to assess because we lack further information on their demographic profile.

### Conclusion

The Infomail open rates and click rates on embedded links were moderate and to an expected extent but were highest for nutrition and prevention topics and in general were dependent on the Infomail’s topic. Infomails were used for stakeholders’ daily work. Our study shows that dissemination of research evidence in a summary format as a targeted email may be most useful for specific stakeholders and should be sent in regular intervals, because recipients rate them as more useful when they remember receiving them. Infomails are a “push” activity and can be used as a possible strategy to support evidence-informed decision-making.

Furthermore, we showed that data, statistics and reports as well as academic research evidence were the information types most frequently used by stakeholders. Scientific journals were indicated by two-thirds of respondents as the preferred way of getting informed about research evidence; thus we can conclude that the surveyed stakeholders prefer to use so-called “pull” activities.

## Data Availability

The data underlying this article will be shared on reasonable request to the corresponding author.

## Supplementary Material

Supplementary Table S1 Number of clicks on links within the Infomail and click rates, per Infomail (Evaluation of Cochrane Public Health Europe Infomails, Austria/Germany/Switzerland, 2020).

Supplementary Table S2 Results of the Infomail campaign reports for all Infomails, per country (Evaluation of Cochrane Public Health Europe Infomails, Austria/Germany/Switzerland, 2020).

Supplementary Table S3 Descriptive information of the online survey sample (Evaluation of Cochrane Public Health Europe Infomails, Austria/Germany/Switzerland, 2020).

Supplementary Table S4 Ways in which the information from the Infomails was used in the context of work (n=244) (Evaluation of Cochrane Public Health Europe Infomails, Austria/Germany/Switzerland, 2020).
