# Supplementary material for: Dissemination of Evidence by Cochrane Public Health Europe in German-Speaking Countries: An Online Stakeholder Survey
Source: Int J Public Health. 2022 Dec 16;67:1605265. doi: 10.3389/ijph.2022.1605265 (PMC9800417; doi:10.3389/ijph.2022.1605265)
Supplement: Supplementary file 1 [file DataSheet1.docx]

# Supplementary material

## Methods: Questionnaire development description

### Survey part 1: Type, frequency and usefulness of information sources, and preferred methods for informing about research evidence

The first question asked participants to indicate what information sources they commonly use in their work. We used the information types and definitions by Zardo et al.^22^ and amended them to fit our purpose: 1) Data, statistics and reports, 2) policies/legislation and legal information, 3) practice guidelines, 4) expertise and advice, 5) academic research evidence and 6) other information sources (with the option to name a further source). Participants could select one or more information type(s) they use in their work. Based on the answers to question 1, they were further asked to estimate their frequency of use only for the information sources they indicated in the previous question. Frequency of use was measured as ‘daily’, ‘weekly’, ‘monthly’, ‘quarterly’, and ‘less than quarterly’. In the third question, respondents were asked to rate the usefulness of the information sources used for their daily work on a five-point Likert scale ranging from ‘very useful’ to ‘not at all useful’. This question was inspired by the survey by Oliver et al.^23^ In the fourth question, participants chose the three most suitable options of preferred ways to inform themselves about research results in the course of their work from a list of 13 possible options and had the option to name a further method (scientific journals, research reports, policy briefs, seminars or workshops [online or in-person webinars], academic and other conferences, personal communication with experts, email alerts, newsletters, personally targeted mailings, press releases, newspaper articles, television or video reports, blog posts, social media [Twitter, Facebook, etc.], others). We used and amended the list of options from Jacob et al.^21^

### Survey part 2: Cochrane Public Health Europe Infomail

In part 2 of the online survey, we first asked whether respondents know the work of Cochrane and how trustworthy they regarded information provided by Cochrane on a five-point Likert scale from ‘very trustworthy’ to ‘not trustworthy at all’. The impact of our Infomails was assessed by asking the respondents in which ways they used the Infomails’ information in the context of their work and provided a list of 10 possible ways. Respondents could choose all that apply. The usefulness of Infomails for their daily work was assessed on a five-point Likert scale from ‘very useful’ to ‘not at all useful’ and included the further option ‘Infomail not received’.

We provided a sample Infomail for each country (Austria, Germany and Switzerland) and subsequently asked respondents to rate the Infomail’s structure, comprehensiveness, information content and length on a five-point Likert scale. In an open-ended question, respondents had the opportunity to give suggestions for improvement or to write anything positive or negative that struck them while reading the sample Infomail.

To receive feedback on how to improve our Infomail service in the future, we asked for interest in certain research topics and other preferred delivery formats.

### Survey part 3: Demographics

The last part of the survey comprised demographic questions related to the person (age and sex) and their work (country working in, work field, position description and years of work experience).

### References (same numbering as in the main document)

21 Jacob RR, Allen PM, Ahrendt LJ, Brownson RC. Learning about and using research evidence among public health practitioners. American journal of preventive medicine 2017;52:S304-S308.

22 Zardo P, Collie A. Type, frequency and purpose of information used to inform public health policy and program decision-making. BMC Public Health 2015;15:381.

23 Oliver K, de Vocht F. Defining ‘evidence’: a survey of public health policy makers’ needs and preferences. Eur J Public Health 2017;27:112-117.

## Email campaign reports

Supplementary Table S1: Number of clicks on links within the Infomail and click rates, per Infomail (Evaluation of Cochrane Public Health Europe Infomails, Austria/Germany/Switzerland, 2020).

| **Infomail number** | **Short title of Cochrane review** | **Link to German PLS/abstract** | | **Link to full text** | |
| --- | --- | --- | --- | --- | --- |
|  |  | **Unique clicks (n)** | **Click rate per unique opening (%)** | **Unique clicks (n)** | **Click rate per unique opening (%)** |
| 1 | Welfare-to-work interventions | 2 | 10.0% | 0 | 0.0% |
| 2 | Unconditional cash transfers | 2 | 8.3% | 0 | 0.0% |
| 3 | Nutritional labelling | 20 | 35.1% | 6 | 10.5% |
| 4 | Multiple risk behaviours | 22 | 36.1% | 8 | 13.1% |
| 5 | Ambient air pollution | 2 | 10.5% | 0 | 0.0% |
| 6 | Environmental interventions to reduce SSB consumption | 27 | 24.8% | 17 | 15.6% |
| 7 | Iodine fortification | 9 | 34.6% | 4 | 15.4% |
| 8 | Altering availability of food products | 24 | 25.8% | 20 | 21.5% |
| 9 | Fortification (5 reviews) | 0 | 0.0% | 1 | 5.3% |
| 10 | Taxation of sugar | 32 | 36.0% | 10 | 11.2% |
| 11 | Video calls to reduce social isolation | 21 | 21.4% | 3 | 3.1% |
| 12 | Travel‐related control measures | 12 | 24.0% | 4 | 8.0% |
| 13 | Interventions to reduce sedentary behaviour | 11 | 18.3% | 3 | 5.0% |
| 14 | Taxation of the fat content of foods | 8 | 11.8% | 4 | 5.9% |
| 15 | Wheat flour fortification with iron | 2 | 13.3% | 0 | 0.0% |
|  | **Median** | **11** | **21.4%** | **4** | **5.9%** |
|  | **Sum** | **194** |  | **80** |  |

Abbreviations: PLS = plain language summary, SSB = sugar-sweetened beverages

Supplementary Table S2: Results of the Infomail campaign reports for all Infomails, per country (Evaluation of Cochrane Public Health Europe Infomails, Austria/Germany/Switzerland, 2020).

| **Country** | | **Number of Infomail recipients (range)** | **Successful deliveries (n) / %** | | **Opens (n) / open rate per successful delivery (%)** | | **Clicks (n) / click rate per unique opening (%) / click rate per successful delivery (%)** | | |
| --- | --- | --- | --- | --- | --- | --- | --- | --- | --- |
| Austria | Median | 51 (19–176) | 50 | 97.4% | 17 | 22.9% | 7 | 35.0% | 9.2% |
| Germany | Median | 47 (15–214) | 44 | 97.8% | 10 | 24.1% | 3 | 30.8% | 7.1% |
| Switzerland | Median | 74 (27–164) | 72 | 97.3% | 20 | 30.4% | 4 | 18.2% | 4.3% |
| **All Infomails** | Median | **56 (15–214)** | **54** | **97.4%** | **14** | **25.0%** | **4** | **28.6%** | **6.3%** |

## Online survey among previous Infomail recipients

Supplementary Table S3: Descriptive information of the online survey sample (Evaluation of Cochrane Public Health Europe Infomails, Austria/Germany/Switzerland, 2020).

|  | **n** | **%** |
| --- | --- | --- |
| *Gender (n=227)* |  |  |
| Female | 122 | 53.7 |
| Male | 95 | 41.9 |
| Non-binary | 1 | 0.4 |
| No answer | 9 | 4.0 |
| *Age (n=227)* |  |  |
| < 30 years | 3 | 1.3 |
| 30–39 years | 30 | 13.2 |
| 40–49 years | 61 | 26.9 |
| 50–59 years | 95 | 41.9 |
| ≥ 60 years | 38 | 16.7 |
| *Country (n=267)* |  |  |
| Germany | 58 | 21.7 |
| Austria | 121 | 45.3 |
| Switzerland | 88 | 33.0 |
| *Field of work (n=227)* |  |  |
| Research | 59 | 26.0 |
| Practice, planning and operational implementation of programmes and projects | 51 | 22.5 |
| Associations and interest groups | 45 | 19.8 |
| Administration, authority: planning and development of strategies or their implementation | 44 | 19.4 |
| Supporting body for political decision-makers or policy advice | 13 | 5.7 |
| Political body: planning and development of strategies, legislation | 8 | 3.5 |
| Other | 7 | 3.0 |
| *Position title (n=227)* |  |  |
| Managerial position (with staff responsibility) | 152 | 67.0 |
| Managerial position (without staff responsibility) | 42 | 18.5 |
| Not in a managerial position | 33 | 14.5 |
| *Work experience (in years) (n=227)* |  |  |
| < 1 year | 4 | 1.8 |
| 1–5 years | 47 | 20.7 |
| 6–10 years | 46 | 20.3 |
| 11–15 years | 47 | 20.7 |
| > 15 years | 83 | 36.6 |
| *Familiarity with Cochrane (n=252)* |  |  |
| Yes | 183 | 72.6 |
| No | 69 | 27.4 |

Abbreviations: n = sample size

Supplementary Table S4: Ways in which the information from the Infomails was used in the context of work (n=244) (Evaluation of Cochrane Public Health Europe Infomails, Austria/Germany/Switzerland, 2020).

|  | **n** | **%** |
| --- | --- | --- |
| Provided me with arguments for discussions | 96 | 39.3 |
| I could use in reports or statements | 62 | 25.4 |
| Served to put issues on the political agenda | 26 | 10.7 |
| Served as an information basis for the content and direction of policies or strategies | 55 | 22.5 |
| Served as a basis of information for the development of programmes or interventions | 50 | 20.5 |
| Served as a information basis for the development of guidelines or requirements for practical work | 32 | 13.1 |
| Served as a source of information for further research work | 35 | 14.3 |
| I passed it on to colleagues at work | 86 | 35.2 |
| Were of no relevance to my daily work | 11 | 4.5 |
| I do not remember having received an infomail | 87 | 35.7 |
| Other ways used the information from the Infomails in the context of work | 4 | 1.6 |

***Abbreviations:*** *n=sample size*
